# Impact of a Multifaceted and Clinically Integrated Training Program in Evidence-Based Practice on Knowledge, Skills, Beliefs and Behaviour among Clinical Instructors in Physiotherapy: A Non-Randomized Controlled Study

**DOI:** 10.1371/journal.pone.0124332

**Published:** 2015-04-20

**Authors:** Nina Rydland Olsen, Peter Bradley, Birgitte Espehaug, Monica Wammen Nortvedt, Hildegunn Lygren, Bente Frisk, Jan Magnus Bjordal

**Affiliations:** 1 Centre for Evidence Based Practice, Faculty of Health and Social Sciences, Bergen University College, Bergen, Norway; 2 Physiotherapy Research Group, Department of Global Public Health and Primary Care, University of Bergen, Bergen, Norway; 3 Public Health Wales, Cardiff, United Kingdom; 4 Department of Physiotherapy, Faculty of Health and Social Sciences, Bergen University College, Bergen, Norway; 5 Department of Physiotherapy, Haukeland University Hospital, Bergen, Norway; Iran University of Medical Sciences, IRAN, ISLAMIC REPUBLIC OF

## Abstract

**Background and Purpose:**

Physiotherapists practicing at clinical placement sites assigned the role as clinical instructors (CIs), are responsible for supervising physiotherapy students. For CIs to role model evidence-based practice (EBP) they need EBP competence. The aim of this study was to assess the short and long term impact of a six-month multifaceted and clinically integrated training program in EBP on the knowledge, skills, beliefs and behaviour of CIs supervising physiotherapy students.

**Methods:**

We invited 37 CIs to participate in this non-randomized controlled study. Three self-administered questionnaires were used pre- and post-intervention, and at six-month follow-up: 1) The Adapted Fresno test (AFT), 2) the EBP Belief Scale and 3) the EBP Implementation Scale. The analysis approach was linear regression modeling using Generalized Estimating Equations.

**Results:**

In total, 29 CIs agreed to participate in the study: 14 were invited to participate in the intervention group and 15 were invited to participate in the control group. One in the intervention group and five in the control group were lost to follow-up. At follow-up, the group difference was statistically significant for the AFT (mean difference = 37, 95% CI (15.9 -58.1), p<0.001) and the EBP Beliefs scale (mean difference = 8.1, 95% CI (3.1 -13.2), p = 0.002), but not for the EBP Implementation scale (mean difference = 1.8. 95% CI (-4.5-8.1), p = 0.574). Comparing measurements over time, we found a statistically significant increase in mean scores related to all outcome measures for the intervention group only.

**Conclusions:**

A multifaceted and clinically integrated training program in EBP was successful in improving EBP knowledge, skills and beliefs among CIs. Future studies need to ensure long-term EBP behaviour change, in addition to assessing CIs’ abilities to apply EBP knowledge and skills when supervising students.

## Introduction

Health care professionals are expected to make evidence-based clinical decisions. Evidence-based practice (EBP) involves integrating the best available research evidence with clinical expertise and patient values, within the context of available resources [1,2]. With the publication of the Sicily statement [1], it became clear that health care professionals should incorporate the necessary knowledge, skills and attitudes of EBP into their training and registration requirements. Curricula should be based on the five EBP steps and processes: asking clinical questions, searching for and appraising research evidence, integrating the evidence into clinical practice and evaluating this process [1]. To ensure that future health care graduates learn how to incorporate EBP steps with their own life-long learning and patient care, EBP should be an integral part of learning throughout their training; including clinical education [1,3].

Clinical education is recognized as an important element in physiotherapy education ([4], p. 125,[5]) and provides an opportunity to learn EBP in authentic clinical settings. Learning in clinical settings requires total immersion to learn the culture and norms of the profession [6]; by actively engaging and participating within the community of practice [7,8]; comprised of physiotherapists, other professionals, other students, patients and their family members [9]. Engagement in the community of practice is also dependent on a formal mentor; the clinical instructor (CI) [10]. The CI is a practicing clinical physiotherapist at the clinical placement site and is responsible for supervising physiotherapy students [5]. Physiotherapists volunteer or are assigned to be CIs. The influence of the CI is recognized as central to students’ knowledge growth and professional development [11,12]. In addition to mentoring and supervising students, the CI should serve as a role model [5]. Results from several studies within different disciplines indicate that students lack role models with strong skills in EBP, in particular among their CIs [13–18]. CIs themselves recognize that they need training in EBP [15,19,20]. The effect of EBP training among undergraduates, postgraduates and practicing health care professionals has been studied extensively, and interventions of varying content, format and duration have been evaluated [21]. Multifaceted interventions that combine teaching strategies, are clinically integrated and involve assessment, lead to improvements in EBP knowledge, skills and attitudes amongst learners [21]. Among practicing health professionals, such interventions also lead to improvements in EBP behaviour [21]. The impact of teaching EBP to CIs has been specifically addressed only in a small number of uncontrolled before-and after studies [22–24]. Hagler et al. [22] tested the impact of an EBP workshop among staff nurse preceptors (CIs), and found that preceptors’ EBP beliefs improved. Kouhpayehzadeh et al. [23] tested the impact of an EBP workshop among clinical teachers in medicine, and found improvements relating to EBP attitudes and skills. Weberschock et al. [24] tested the effect of a web-based course on how to teach evidence-based medicine principles, and found that knowledge improved among clinical teachers in medicine. In addition to these uncontrolled before-and-after studies, Mohide & Matthew-Maich [25] piloted and evaluated an EBP workshop among nursing preceptor-student pairs. Telephone interviews indicated improvements with regard to EBP attitudes, skills, and sharing of EBP knowledge and skills with colleagues [25]. To the best of our knowledge, no controlled studies have been conducted to study the impact of teaching EBP to CIs. The aim of this non-randomized controlled study was to assess the short and long term impact of an EBP program on the knowledge, skills, beliefs and behaviour of CIs supervising physiotherapy students.

## Methods

### Design and participants

We conducted a non-randomized controlled study, with a six-month follow-up, to assess the impact of an EBP program on CIs’ EBP knowledge, skills, beliefs and behaviour. Self-administered questionnaires were used among CIs before and after the intervention, and at six-month follow-up. The study was carried out from September 2008 to November 2009. Exemption from obtaining ethical approval was granted by the Regional Committee for Medical and Health Research Ethics, Western-Norway. The study was approved by the Norwegian Social Science Data Services (NSD). Written informed consent was obtained prior to the intervention. The study was supported by all of the involved institutions: Bergen University College (BUC), Haraldsplass Deaconess Hospital (HDS) and Haukeland University Hospital (HUS).

BUC is one of four university colleges in Norway that offers a three-year bachelor program (180 ECTS-credits) in physiotherapy [26,27]. During the three-year bachelor program, physiotherapy students spend 30 weeks in clinical work, in various clinical settings: primary health care, outpatient clinics, rehabilitation clinics, local hospitals and university hospitals. During clinical placement, students are supervised by CIs who are physiotherapists practicing at the clinical placement site.

At the start of the study (August 2008), 37 physiotherapists at different hospitals in Norway were assigned the role as CIs for 3^rd^ year physiotherapy students (BUC), for the students’ final 10-week clinical placement in January 2009. These CIs (n = 37) were asked to volunteer for the study via e-mail.

**Fig 1:**
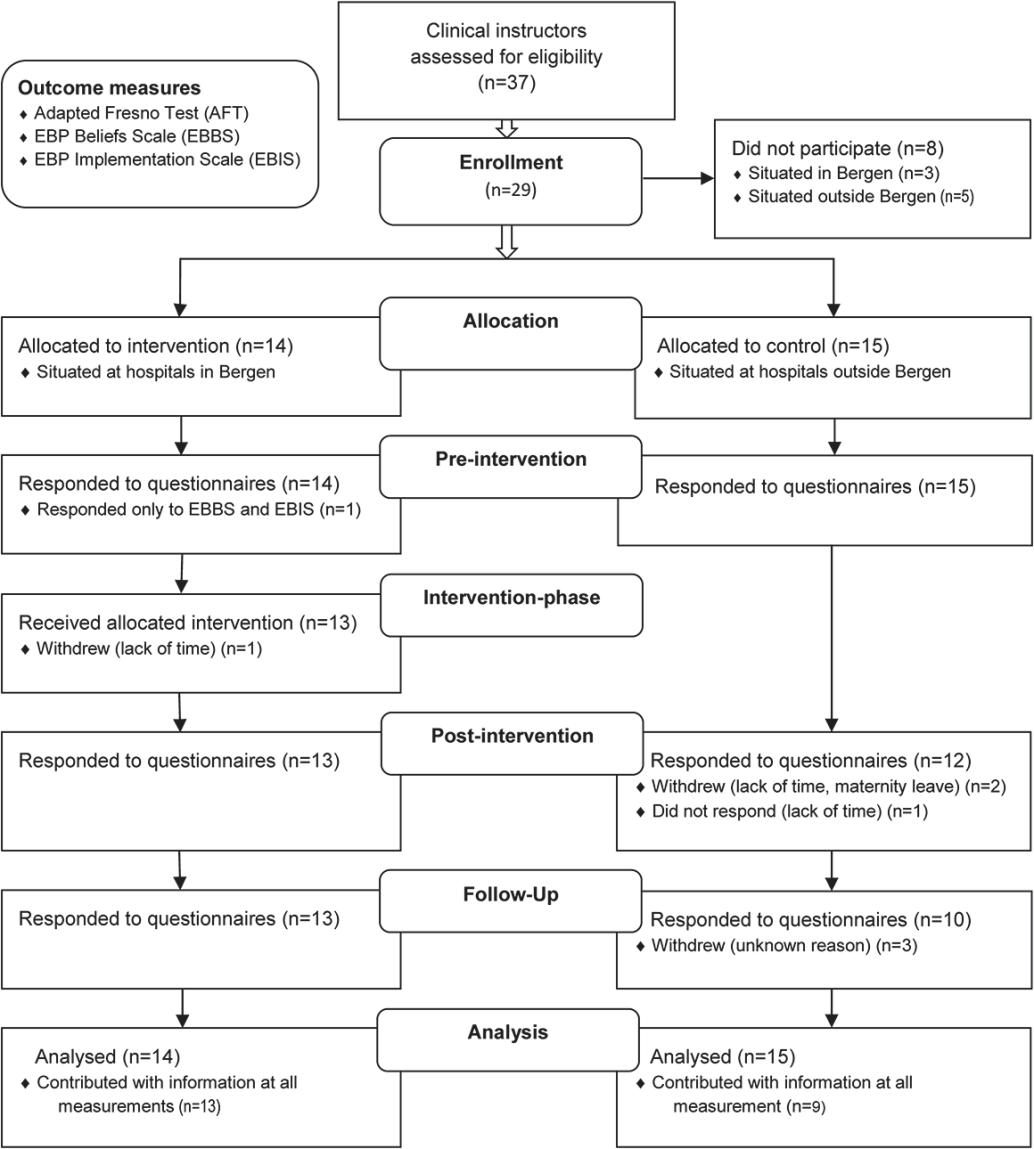
Flow diagram of study.

We invited CIs at hospitals situated in Bergen to participate in the intervention group (n = 17), and CIs at hospitals situated outside Bergen to participate in the control group (n = 20) (Fig 1). This decision was based on consideration of time and cost of travelling. Hospitals outside Bergen are situated far apart, and the geographical distances between Bergen and the hospitals situated outside Bergen can be fairly large (up to 400 km), and often only one or two CIs are situated at these hospitals.

### The intervention

#### Intervention content and delivery

The intervention consisted of a multifaceted and clinically integrated training program in EBP (6 ECTS-credits), delivered to CIs over a six-month period (October 2008- April 2009) (Table 1). The training program was multifaceted as combinations of several teaching strategies were employed: workshops, assignments, supervision and exams. The workshops were a mixture of lectures (didactic sessions) and small-group activities that required participants to be interactive. Four half-day workshop sessions covering the EBP steps and processes were held in classrooms at hospital settings (HUS) and delivered sequentially over a six-week period (Table 1). Between and after workshops, five individual written assignments (Week 2, 4, 7, 1, 21) were required from the participants. To ensure clinically integrated learning of EBP, each assignment required participants to reflect on and describe how to apply the EBP steps in real clinical situations. Assignment one and two required participants to write a coherent paper on identified clinical information need and question formulation. For the three first assignments they had to reflect on how to supervise students in using the EBP steps. Assignment three to five required participants to work through all EBP steps using the EBP tool (Table 2). The EBP tool can be used to document the EBP steps and process: information need, clinical question asked, search strategy and result of this, critical appraisal of the research results found (validity, reliability and applicability), if and how the research results are integrated or shared in clinical practice and how the changes made to clinical practice are evaluated. The EBP tool is a learning tool intended to provide health care professionals with practical EBP skills. The EBP steps and processes registered in one document facilitate the learning process and the possibility of receiving and giving feedback. Development of this tool was inspired by working files developed for trainee doctors in Norway [28]. For each assignment, participants received supervision via phone and/or email, in addition to guidance from a librarian when necessary. All five assignments had to be completed before the final exam. The exam took form of an individual oral presentation, where participants focused on how to apply the EBP steps to a real patient situation and how to supervise students in the EBP process (Week 27) (Table 1). The exam was assessed as pass/fail. The objectives of the program (Table 1) were related to the EBP steps and processes described previously [29].

**Table 1:** Training program in evidence-based practice (EBP): workshops, assignments and objectives (6 ECTS-credits^a^).

Weeks:	1–2	2–4	5	6	7	13	16	18	21	27
**Workshops/ meetings**	**1** ^**st**^ **Workshop** Introduction to EBP concepts: What is EBP, the five EBP steps, information sources, question formulation, theory on how to supervise students in clinical placements.	**2** ^**nd**^ **Workshop** Introduction to research methods; qualitative and quantitative.	**3** ^**rd**^ **Workshop** Introduction to search strategy and relevant databases such as PubMed, Pedro, Cochrane Library and other pre-appraised sources of evidence.	**4** ^**th**^ **Workshop** How to read an article and critical appraisal; randomized controlled study (RCT).		**1** ^**st**^ **Meeting** Discussion of different challenges, including assignments (1 hour).		**2** ^**nd**^ **Meeting** Discussion of different challenges, including assignments and exam (1 hour).		
**Assignments/ exams**	**1** ^**st**^ **Assignment** Consider and reflect upon clinical situations where you had an information need. Discuss how to supervise students in identifying information need (700–1000 words).	**2** ^**nd**^ **Assignment** Describe relevant core questions; use PICO^b^ to formulate a precise clinical question; discuss relevant study designs; including, advantages and disadvantages. Discuss how to supervise students in EBP step 1 (relate to real situations) (700–1000 words).			**3** ^**rd**^ **Assignment** Read and critically appraise a RCT study. Use the EBP tool to describe and reflect upon actions for each EBP step, including how to supervise students/share with colleagues. No word limit.		**4** ^**th**^ **Assignment** Identify a real information need from your clinical practice and work through all EBP steps. Use the EBP tool. No word limit.		**5** ^**th**^ **Assignment** Identify a real information need from your clinical practice and work through all EBP steps. Use the EBP tool. No word limit.	**Exam**: One day seminar with 10 minutes individual oral presentations. **Exam topic:** Illustrate how to work through all EBP steps. Use the EBP tool and illustrate how to supervise students in the EBP steps and processes.
**Objectives**	**EBP step 1:** Identify information need; reflect on how to apply this skill to clinical practice and in supervision.	**EBP step 1:** Formulate PICO^b^ questions pertinent to clinical area. Gain a basic understanding of different study designs; differentiate between which designs that best answers different core questions.	**EBP step 2:** Search for research evidence in relevant databases for health and social care professionals.	**EBP step 3:** Critically appraise research evidence for validity, relevance and applicability.	**EBP step 1–5:** Apply all five steps to a hypothetical scenario; reflect on how to apply the evidence in clinical practice and in supervision.		**EBP step 1–5:** Apply all five steps to a real clinical scenario; reflect on how to apply the evidence in clinical practice and in supervision.		**EBP step 1–5:** Apply all five steps to a real clinical scenario; reflect on how to apply the evidence in clinical practice and in supervision.	**EBP step 1–5:** Apply all five steps to a real clinical scenario; reflect on how to apply the evidence in clinical practice and in supervision.

^a^ECTS-credits = European Credit Transfer and Accumulation System,

^b^PICO:“P” = patients, “I” = intervention, “C” = control or comparison, and “O” = outcome

**Table 2:** The evidence-based practice (EBP) tool: tasks and questions.

	The EBP tool
**Information need**	What topic do you need information on?
	What do know about this topic today?
	What is usual practice concerning this topic today.
**Question formulation**	Formulate your question.
	Fill in the different PICO^a^ elements.
**Literature search**	Describe and document your search strategy:
	• Which source/database(s) did you use?
	• Explain your choice of source/database.
	• Which search terms did you use?
	• How did you combine the search terms?
	• Attach/copy and paste the search strategy.
	• Where did you find relevant articles?
	Choose one article and explain your rationale for this.
	Describe your experiences with the search.
	Attach the article (and/or summary); copy and paste the internet link.
**Critical appraisal**	What was the research question in the article?
	What kind of clinical question was this?
	What kind of study design was applied?
	Are the results valid?
	What were the results?
	Can you apply the results to your clinical practice?
**Implementation**	With regard to the question you have formulated; What conclusions can you draw?
	Write a short summary of the results in the article. Based on this; What suggestions do you have for how to integrate the evidence with your clinical practice?
	Reflect on how you could share this knowledge with your students and colleagues.
**Evaluation**	If you changed your practice, how will you evaluate this?
**Supervision**	Did you need supervision? Please describe the supervision you needed.
**Time**	How long time did it take you to work through the steps?

^a^ PICO:“P” = patients, “I” = intervention, “C” = control or comparison, and “O” = outcome

The overall idea behind the intervention was that learning of EBP should be integrated into clinical workplaces; a thought that is in line with different experiential learning theorists, in particular socio-cultural theorists such as Vygotsky, Lave and Wenger [30]. The intervention was based on several aspects from these latter theorists, by viewing learning as situated and triggered by authentic practice-based experiences, and considering interaction fundamental to learning [7,30,31].

The workshop delivery was inspired by the Critical Appraisal Skills Program (CASP) where workshops are problem-based, small-group oriented, enjoyable, grounded within the clinical decision making process, and focus is on interactive teaching in a safe environment and use of high-quality and user-friendly materials [32–34].

The program was delivered by a project group of five physiotherapists (including NRO, HL, BF), from both academic and clinical positions, and with a range of expertise in EBP, physiotherapy, higher education and research.

### Data collection

#### Outcome measures

We chose to focus on outcome measures defined by Shaneyfelt et al. [35] as the EBP domains for evaluating EBP in education, including knowledge, skills, attitudes and behaviour. Knowledge is defined as knowledge about EBP (e.g. EBP steps), whereas skills is defined as applying knowledge about EBP by performing EBP steps to solve clinical scenarios, for example with a written patient cases (e.g. formulating a clinical question or finding the best evidence). Attitude is defined as attitudes towards EBP (e.g. beliefs about the value of EBP). EBP behaviour is defined as actual performance of EBP in practice, as in enacting of the EBP steps in practice, performing evidence-based maneuvers in actual practice (e.g. following guideline prescriptions) or affecting patient outcomes. In this study, we focused on the EBP behaviour as in self-reported enacting of the EBP steps in practice.

For evaluating these four different EBP domains a wide range of instruments were identified in a systematic review by Shaneyfelt et al [35]. However, no valid instruments were identified for evaluating EBP in educational interventions among rehabilitation professionals. We were able to identify only three previously validated questionnaires instruments for assessing the impact of the educational program of this study: 1) The Adapted Fresno test (AFT) [36,37], 2) the EBP Belief Scale [38], and 3) the EBP Implementation Scale [38].

Permission to translate and use these three different instruments was obtained from the respective authors [36,38]. The AFT, the EBP Belief Scale and the EBP Implementation Scale were translated to Norwegian using a forward and backward translation procedure as described by the World Health Organization [39]. In addition, we collected demographic data such as age and gender, and background information about type and size of position, postgraduate education and years of experience.

#### The Adapted Fresno test (AFT)

The AFT is a seven-item test developed for rehabilitation professionals, educators and researchers to measure change in the EBP skills and knowledge (EBP competence) following training in EBP [36]. The AFT measures EBP knowledge about: information sources, the hierarchy of evidence, the study design that best answers questions about effectiveness, keywords and limits to use when searching and methodological biases in study designs. The AFT measures EBP skills related to: the ability to write a focused clinical question, the ability to reflect upon advantages and disadvantages of information sources, the ability to describe an effective and efficient search strategy and the ability to interpret and critically appraise a published paper. The AFT is focused around different clinical scenarios relevant to rehabilitation professionals. There are three versions of the AFT that include identical items, but different sets of clinical scenarios to help minimize practice effects when AFT is used for pre-, post- and follow-up testing [36]. The total score range from 0–156, and the test takes 20 minutes to complete and 20 minutes to score using a scoring matrix developed by McCluskey and Bishop [36]. The AFT has been reported to have acceptable psychometric properties, with excellent inter-rater reliability for AFT total score (ICC > 0.9) and acceptable internal consistency (Cronbach’s alpha 0.74) [36]. Improvements of 10% (15.6 points) in the mean total score at post-workshop, and 15% (23.4 points) at follow-up are considered as educationally important change, when compared to baseline [37]. The AFT is most useful for evaluating change in novice learners [36].

#### The EBP beliefs scale

The EBP beliefs scale was “…designed to measure clinicians’ beliefs about the value of EBP and their beliefs/confidence in implementing it in practice” ([38], p. 209); and Melnyk et al. found that the scale was sensitive to a wide range of attitudes ([38], p. 214). Melnyk et al. ([38], p. 210]) defined EBP beliefs as “…endorsement of the premise that EBP improves clinical outcomes and confidence in one’s EBP knowledge/skills”. The test contains 16 statements addressing EBP beliefs on a continuum from 1 (strongly disagree) to 5 (strongly agree) (5-point Likert scale). Negatively phrased items (item 11 and 13) are reversed before summing responses of the 16 items, with a total score that ranges between 16 and 80 [38]. Testing of the EBP Beliefs scale has shown that it has excellent internal consistency (Cronbach alpha 0.9) and measures a unidimensional construct [38].

#### The EBP implementation scale

The EBP Implementation Scale is designed to measure clinicians’ implementation of essential components and steps of EBP [38]. Melnyk et al. ([38], p. 210) defines EBP implementation as “engaging in relevant behaviours, including: (1) seeks and appraises scientific evidence, (2) shares evidence or data with colleagues or patients, (3) collects and evaluates outcome data, and (4) uses evidence to change practice.” The test contains 18 statements where participants are asked to indicate how often in the past eight-weeks they performed the item on a 5-point frequency scale from 0 (“0 times”) to 4 (>8 times). Scoring the 18-item test involves summing responses, and a total score could range from 0 to 72. Testing of the EBP Implementation scale has shown that it has excellent internal consistency (Cronbach alpha 0.96) and measures a unidimensional construct [38].

#### Data collection procedure

All participants were asked to individually complete the paper and pencil versions of the EBP Beliefs scale, the EBP Implementation Scale and the AFT at three different measurement times: at pre-intervention in September 2008, at post-intervention in May 2009 and at follow-up in November 2009. In addition, all participants were asked to fill out demographic sheets at pre-intervention (September 2008). Questionnaires and demographic sheets from the control group were collected via mail. Participants in the intervention group were gathered at a meeting room at the hospital (HUS) where they filled out the questionnaires.

The AFT tests were scored during a two-week period. A training session was conducted using examples of scored and unscored copies, a similar procedure as described by McCluskey and Bishop [36]. All the AFTs (n = 76) were scored independently by two raters with a good understanding of EBP (first author, and a nurse (PhD) experienced with teaching EBP). Both raters were blinded to the status of the AFTs, as to whether the tests were from the control or the intervention group. To measure agreement between raters we calculated intraclass correlation coefficients (ICC) type 2, 1 (random effect model) [40]. Agreement between raters was very good for all the total AFT scores (Version 1: ICC 0.89, 95% CI 0.74–0.95; Version 2: ICC 0.95, 95% CI 0.88–0.98; Version 3: ICC 0.97, 95% CI 0.92–0.99). For the analysis we used AFT consensus scores; disagreement among raters was solved by discussing question-by-question.

#### Data analysis

IBM SPSS Statistics version 20 (SPSS Inc., Chicago, USA 2012) was used for data analyses. Descriptive statistics, including frequencies, percentages, mean, range and standard deviation (SD) were calculated to describe characteristics of the participants. To account for correlated data imposed by the study design with repeated measures of the outcome, we used generalized estimating equations (GEE) ([41], p. 62–77) to estimate differences in mean scores. An interaction term between group and measurement time was included in the regression models to investigate time dependent group differences. In these analyses, an unstructured working correlations structure was applied and standard errors were calculated using robust estimates. Additional analyses were performed to adjust for potential confounding by age, gender, type of position, size of position, type of post-graduate education and years of experience. Estimated differences in outcomes were reported as mean difference (MD) with 95% confidence interval (95% CI). P-values less than 0.05 were considered statistically significant for all analyses.

Internal consistency for all outcome measures was calculated using Cronbach’s α. Alpha values of ≥ 0.70 were regarded as satisfactory for comparing groups [42].

## Results

### Participants

From a total of 37 eligible CIs, 29 (78.4%) chose to participate in this study (Fig 1). Fourteen CIs were allocated to the intervention group and 15 to the control group. In total, 13 from the control group and nine from the intervention group contributed with information at all measurements.

Table 3 provides an overview of the participants’ characteristics. The intervention group and the control group were similar at baseline with respect to all participant characteristics. The majority were female (n = 26) and the mean age was 39.7 years (range 26–61, SD 9.9). Mean years of experience was 12.9 years (range 2–32, SD 8.6). More than half of the participants had some kind of postgraduate education. Only three of the participants held a leadership position, and nine of the participants were specialists. Only one participant was in a part-time position, and the rest of the participants held 80–100% positions.

**Table 3:** Characteristics of participants (N = 29).

Characteristics (n)	Intervention group (n = 14)	Control group (n = 15)
Age (years)		
20–29	2	4
30–39	3	5
40–49	7	4
> 50	2	2
Mean (range, SD^a^)	40.5 (26–55, 8.6)	38.9 (26–61, 11.2)
Gender		
Female	12	14
Male	2	1
Type of position (in addition to physiotherapist)		
Leader	1	2
Specialist	4	5
Responsible for professional development	0	1
Size of position		
80–100%	14	14
50%	0	1
Postgraduate education		
Yes	8	9
No	6	6
Postgraduate education (type)		
Master of Science	0	1
Postgraduate course in research methods	3	1
Postgraduate course in EBP	1	1
Other postgraduate courses	4	5
No postgraduate education	6	6
Years of experience as physiotherapist		
0–4 years	1	3
5–9 years	5	5
>10	8	7
Mean (range, SD)	13.6 (2–28, 8.3)	12.2 (2–32, 9.0)

^a^SD = standard deviation

### Internal consistency

Internal consistency measured by Cronbach’s alpha at pre-intervention was satisfactory for all instruments, including the EBP Beliefs scale (0.85), the EPB implementation scale (0.85) and the AFT (0.93).

### Changes in scores related to Adapted Fresno Test, EBP Beliefs and Implementation scale

The GEE regression analyses showed statistically significant differences in favor of the intervention for all three outcome measures at post-intervention. At follow-up, the group difference was statistically significant for two of the outcome measures: the AFT (mean difference = 37, 95% CI (15.9–58.1), P *<*0.001) and the EBP Beliefs scale (mean difference = 8.1, 95% CI (3.1–13.2), P = 0.002) (Table 4).

**Table 4:** Adapted Fresno Test, EBP Beliefs Scale and EBP Implementation scale mean scores in the intervention and the control group.

	Pre-intervention	Post-intervention	Follow-up	MD within groups^a^	95% CI^b^	P-value
**Adapted Fresno Test (range 0–156)**						
Intervention	43.4	80.5	69.4	26.0	(17.5–34.5)	<0.001
Control	34.5	37.5	32.5	-2.0	(-13.7–9.7)	0.740
MD between groups^c^	9.0	43.0	37.0			
95% CI	(-6,3–24,3)	(29,7–56,4)	(15.9–58.1)			
p-value	0.248	<0.001	<0.001			
**EBP Beliefs Scale (range 16–80)**						
Intervention	44.8	52.6	53.6	8.7	(5.6–11.9)	<0.001
Control	43.6	44.7	45.4	1.9	(-1.8–5.5)	0.321
MD between groups	1.2	7.9	8.1			
95% CI	(-2,6–5)	(4–11,8)	(3.1–13.2)			
p-value	0.526	<0.001	0.002			
**EBP Implementation Scale (range 0–72)**						
Intervention	7.7	17.7	12.3	4.6	(1.7–7.5)	0.002
Control	8.9	7.0	10.5	1.7	(-4.2–7.5)	0.574
MD between groups	-1.1	10.7	1.8			
95% CI	(-5–2.8)	(6.4–15)	(-4,5–8.1)			
p-value	0.570	<0.001	0.574			

^a^MD within groups = estimated mean difference between scores at follow-up and pre-intervention

^b^CI = confidence interval

^c^MD between groups = estimated mean difference between scores in the intervention and the control group

All estimates were calculated with GEE regression with adjustment for gender and years of experience.

Comparing measurements over time within groups, we found a statistically significant increase in mean scores related to all outcome measures for the intervention group only. The GEE analysis was adjusted for gender and years of experience. Further adjustment for age, type of position, size of position and type of post-graduate education gave only marginal differences. Total observations from the participants included in the GEE analysis from the intervention group were 40 and 37 from the control group (Fig 1).

## Discussion

This study has generated unique findings on the impact of a multifaceted and clinically integrated training program in EBP among CIs at hospitals in Norway. At follow-up we found statistically significant between-group differences in favor of the intervention group with regard to EBP knowledge, skills and beliefs, but not for behaviour. When comparing measurement over time, a statistically significant increase in mean scores, with regard to knowledge, skills, beliefs and behaviour was found for the intervention group only.

Our findings concur with results from previous systematic reviews on the effect of teaching EBP; concluding that interactive, multifaceted, and clinically integrated interventions that involve assessment lead to improvements in EBP knowledge, skills, attitudes and behaviour among health professionals [21]. This is in line with the socio-cultural perspective on learning [7,8], which support that learning takes place in authentic settings (clinically integrated) and through social interaction (interactive). Previous reviews on the impact of teaching EBP did not identify or include studies specifically focusing on CIs [21]. However, the impact of EBP training on CIs has been evaluated positively in three uncontrolled before-and-after studies within nursing and medicine [22–24]. Findings from these studies have to be interpreted with caution due to methodological deficits (e.g. uncontrolled studies). Furthermore, these studies assessed knowledge and attitudes, not behaviour [22–24]. This is not unusual as many studies on the impact of teaching EBP focus only on outcomes such as attitudes (beliefs about the value of EBP), beliefs/confidence in ability to conduct EBP (self-efficacy), knowledge or skills [2]. However, when investigating the impact of teaching EBP—it is also essential to assess EBP behaviour, as we need to know if learners apply their skills in actual practice [2,35,43]. Assessing only EBP attitudes or beliefs is not sufficient, as EBP attitudes do not necessarily lead to EBP behaviour [44]. Authors of a recent systematic review question the link between attitudes and behaviour, as they identified studies reporting of physiotherapists with positive attitudes failing to implement EBP [44]. In our study, we assessed all the typical outcome measures, including EBP behaviour and found statistically significant between-group differences at post-intervention in favor of the intervention group for all outcome measures, but changes related to behaviour were not sustained at six-month follow-up. After the intervention-phase these CIs might have experienced that they were left alone, without requirements to apply EBP and the support that they received during the intervention phase. Possibly, they were also faced with barriers that hindered them applying EBP. In this study, we did not aim to identify potential EBP barriers at individual and organisational level during or after the intervention. However, lack of time, inability to understand statistics, lack of support from employer, lack of resources, lack of interest, and lack of generalisation of results have been identified as frequently reported barriers among physiotherapists [45]. These barriers could explain why EBP behaviour differences were not sustained in our study.

It is essential to find ways of ensuring long term change in EBP behaviour among CIs, in particularly when considering the potential impact that behaviour among CIs can have on students’ professional development. Cole and Wessel [46] found that CIs demonstrating professional behaviour related to EBP enhanced students learning experience in clinical placements. A non-evidence-based culture and CIs not practicing evidence-based at the placement has been reported as barriers towards implementation of EBP among undergraduate health care students [14,15,17,29]. In contrast, CIs applying the principles of EBP encourage students to practice evidence-based [13,14]. No doubt, CIs are important role models for students and influence students’ visions of how to practice physiotherapy in the future ([4], p. 131). Also, when both students and CIs have EBP competence, there is room for dialogue, for example about real patient situations where students and CIs can discuss the applicability of research evidence. Dialogue is essential for shared meaning to develop [6], and with this type of dialogue CIs and students could develop a shared meaning with regard to how to practice evidence-based. Developing shared meaning about how to practice evidence-based could enable students to make decisions about which behaviours to imitate and develop visions about their own professional identity (i.e. how they want to practice as future professionals) [6].

Few studies have, however, explored if and how EBP is taught or emphasised by clinicians during clinical education [19,20,47]. Kljakovic et al. [20] report of a small percentage of clinicians with training in evidence-based medicine (EBM), and they report of small percentages that teach it. Not surprisingly, clinicians with training were also more likely to teach EBM [20]. Meyer and Willett [47] explored which learning activities physical therapy students reported that their CIs used to promote the acquisition of different core competencies, including employing evidence-based practice. Students reported that promoting the EBP competencies, through skills such as applying quality improvement and utilizing informatics, was one of the learning activities that their CIs did not emphasise to a great extent [47]. Few examples of CIs teaching EBP could be explained by barriers that CIs experience in implementing EBP among students. Hankemeier et al. [48] report of barriers among CIs within athletic training, such as time, equipment, access to literature, knowledge, negative attitudes among colleagues, poor integration between the clinical setting and the classroom, and EBP not integrated across the athletic training program. Future studies on the impact of teaching EBP among CIs need to take these barriers into account.

Our EBP program also focused on how to supervise students in EBP, and CIs participating in this program had to reflect on how they applied EBP steps and principles when supervising students. However, we did not specifically assess if and how the CIs’ transferred their EBP knowledge and skills to supervisory situations with students. Similarly, this has not been assessed in other previous studies on the impact of teaching EBP to CIs. Future studies on the impact of teaching EBP to CIs need to consider if CIs are able to transfer their EBP knowledge and skills to supervisory situations with their students. However, according to Walczak et al. [49] there is a lack of assessment tools for this type of outcome as per today. Therefore, there is a need for developing assessment tools for measuring CIs’ teaching skills and skills related to supervising students in EBP.

### Limitations

This study is the first to assess an interactive and clinically integrated training program in EBP among CIs in physiotherapy education. The controlled design allowed us to efficiently pilot the EBP program and the educational material, including the EBP tool. This study has, however, several limitations. The feasibility of implementing this training program in EBP at other hospitals in Norway, or at hospitals outside Norway, remains uncertain. We conducted a controlled non-randomized study. In this study, true randomization was not performed, mainly because of geographical distances. Blinding of researchers and participants was not possible as the tutors involved in the intervention and the CIs were aware of the allocation. Communication between CIs in the control group and the intervention group may have occurred, although allocation within different geographical areas (hospitals situated in Bergen and outside Bergen) most likely protected against contamination. There may have been a selection bias; CIs who volunteered to participate in the study could have been more motivated than those who did not volunteer. Possibly, our findings could also be biased due to the fact that most of the participants in the intervention group worked at a university hospital; and it has been documented that physiotherapist in such setting feel part of a research-oriented culture [50,51]. Still, there were now statistically significant differences in scores between the groups at pre-intervention. Participants in both the control and the intervention group can be classified as novice learners, reflected by pre-intervention scores below 50% (78/156) for both groups [36].

The limited number of available participants led to low statistical power causing a probable type I error. Although, the sample size limits the statistical power of the study and probably cause low precision, our results showed statistically significant changes on the 5% level for all pre- and post-intervention comparisons, indicating that the impact of training is reasonably large. With regard to the AFT, the reported mean change between groups was 37 points at follow-up when compared to baseline, which is considered an educationally important change [37]. The impact of the intervention was assessed using outcome measures that had been previously validated and psychometrically tested (for this purpose). The fact that change was detected in this study indicates that each of these instruments are responsive to change, also among CIs in Norway. Still, these findings must be interpreted with caution; we need to question if change detected regarding EBP behaviour was reflected in real practice, as we measured self-reported EBP behaviour. Eccles et al. [52] argue that intention (self-reported measures) appears to be a valid proxy measure for behaviour for use in the development of implementation interventions, as measuring actual behaviour is challenging. Still, it might be worth the effort. As pointed out by Shaneyfelt et al. [35], EBP behaviour documented through retrospective self-report might be biased as respondents tend to overestimate their actions. Ideally, behaviour should be measured using some form of activity monitoring [2]; a consideration for future research. Translation of EBP knowledge, skills and attitudes or beliefs to real-time EBP behaviour could be explored, for example by observing and audiotaping practitioners and their interactions with students [44,53,54].

Scoring of the AFT was reliable indicated by excellent ICC values. The outcome assessors were blinded to the status of the AFTs, as to whether the tests were from the control or the intervention group. Assessors were however not blinded when analyzing data related to the EBP Beliefs Scale and the EBP Implementation Scale.

## Conclusions

In this study, we have demonstrated the success of an interactive, multifaceted and clinically integrated training program in EBP among CIs at hospitals in Norway. The intervention resulted in statistically significant increase in mean scores, with regard to knowledge, skills, beliefs and behaviour for the intervention group, when comparing measurement over time. At follow-up, we found statistically significant between-group differences with regard to EBP knowledge, skills and beliefs in favor of the intervention group. Future research needs to investigate how to ensure long-term EBP behaviour change. Further investigations are also needed to investigate whether similar finding can be achieved among other health professionals, in addition to the feasibility of implementing a similar program among a larger group using a stronger study design. Future studies also need to consider CIs’ abilities to transfer their EBP knowledge and skills to supervisory situations with their students.
